# Preparing a healthcare workforce for geriatrics care: an Interprofessional team based learning program

**DOI:** 10.1186/s12877-021-02456-8

**Published:** 2021-11-16

**Authors:** Shelley B. Bhattacharya, Stephen Jernigan, Myra Hyatt, Dory Sabata, Shane Johnston, Crystal Burkhardt

**Affiliations:** 1 University of Kansas Medical Center, School of Medicine, 3599 Rainbow Blvd, Kansas City, Missouri 66160 USA; 2 University of Kansas Medical Center, School of Health Professions, 3901 Rainbow Blvd, Kansas City, Missouri 66160 USA; 3 University of Kansas Medical Center, School of Social Welfare, 3901 Rainbow Blvd, Kansas City, Missouri 66160 USA; 4 University of Kansas, School of Pharmacy, 3901 Rainbow Blvd, Kansas City, Missouri 66160 USA

**Keywords:** Workforce preparation, Geriatrics care, Healthcare systems, Interprofessional education, Team based learning

## Abstract

**Background:**

Improving the care of older adults in our healthcare system involves teams working together. As the geriatrics population rises globally, health science learners need to be prepared to work collaboratively to recognize and treat common conditions in geriatrics. To enable workforce preparation, the Institute of Medicine and the National League for Nursing emphasize the need to implement interprofessional active learning activities for undergraduate healthcare learners at academic medical centers.

**Methods:**

The Geriatrics Champions Program was a team-based learning activity created to meet this task. It was a 24-month program, repeated twice, that impacted 768 learners and 151 faculty from medicine, occupational therapy, physical therapy, nursing, social welfare, psychology, pharmacy and dietetics. Each class was intentionally divided into 20 interprofessional teams that met four times annually. Each session focused on one geriatrics domain. The objectives were centered around the specific geriatrics competencies for each health profession, divided into the eight domains written in the “American Geriatrics Society IM-FM Residency Competencies”. Evaluation consisted of individual and team Readiness Assessment Tests (iRAT and tRAT). Surveys were also used to collect feedback using a Likert scale. Wilcoxon signed rank tests were used to compare iRAT and tRAT scores. Other analyses identified characteristics associated with tRAT performance group (Unpaired t-tests) and tRAT performance on the raw scale (Pearson correlation). Paired t-tests using a 7-level Likert Scale measured pre-post change in learner knowledge.

**Results:**

Student tRAT scores were 30% higher than iRAT scores (*p* < 0.001). Teams were more likely to score 100% on the initial tRAT attempt if more team members attended the current session (*p* < 0.001), more health professions were represented by team members in attendance (*p* = 0.053), and the team had a better track record of past attendance (*p* < 0.01). In the post-program evaluation, learners felt this program was helpful for their career preparation in interprofessional geriatrics care.

**Conclusions:**

Learners understood that teams performed better than individuals in the care of older adults. Feedback from the learners and faculty was consistently positive and learners felt better prepared for geriatrics care. The program’s benefits may extend beyond individual sessions.

## Background

Preparing the workforce to work collaboratively in the care of older adults is an essential component to our healthcare system. Teaching geriatrics competencies to health profession learners is a necessary, but challenging, task. There is both a limited amount of time dedicated to teaching geriatrics and a limited proportion of clinician educators proficient in teaching geriatrics care. In geriatrics, interprofessional education (IPE) models have been used internationally for geriatric syndromes. Dementia care management, teaching the “5 M” Framework outlined by the American Geriatrics Society, palliative care, falls risk assessments, and many other geriatric syndromes have been presented using an IPE model [1–4]. Most were performed during the clinical training years to students and faculty, after the didactic years, or as a one-workshop session. This program intervention adds to the current literature by providing a platform for a two-year didactic curriculum to provide continuous exposure to geriatrics care for interprofessional learners at the graduate-school level.

As our population matures, it becomes increasingly important for learners to recognize geriatrics syndromes, understand who their team players are and treat accordingly with optimal outcomes. The Institute of Medicine’s 2003 report and the National League for Nursing’s 2015 report emphasized a need to implement interprofessional active learning activities for undergraduate healthcare learners at academic medical centers [5, 6]. The Geriatrics Champions Program was designed to create an inclusive interprofessional education (IPE) activity to introduce geriatrics competencies to six professions at an academic medical center with the goal of geriatrics care workforce preparation. The team-based learning (TBL) format was the foundation for promoting learner accountability, stimulating group interaction, and logistically managing large groups of learners. The five-year program impacted 768 learners and 151 faculty (Table 1) from medicine, occupational therapy, physical therapy, social welfare, psychology, nursing, pharmacy and dietetics (Table 2). Priorities for the program were to provide an active learning modality for learner engagement in geriatrics, a chance to practice team collaboration to mimic the workforce and an opportunity for learner and faculty scholarship. Post-program evaluation written comments were reviewed but no formal qualitative analysis was completed.

**Table 1:**
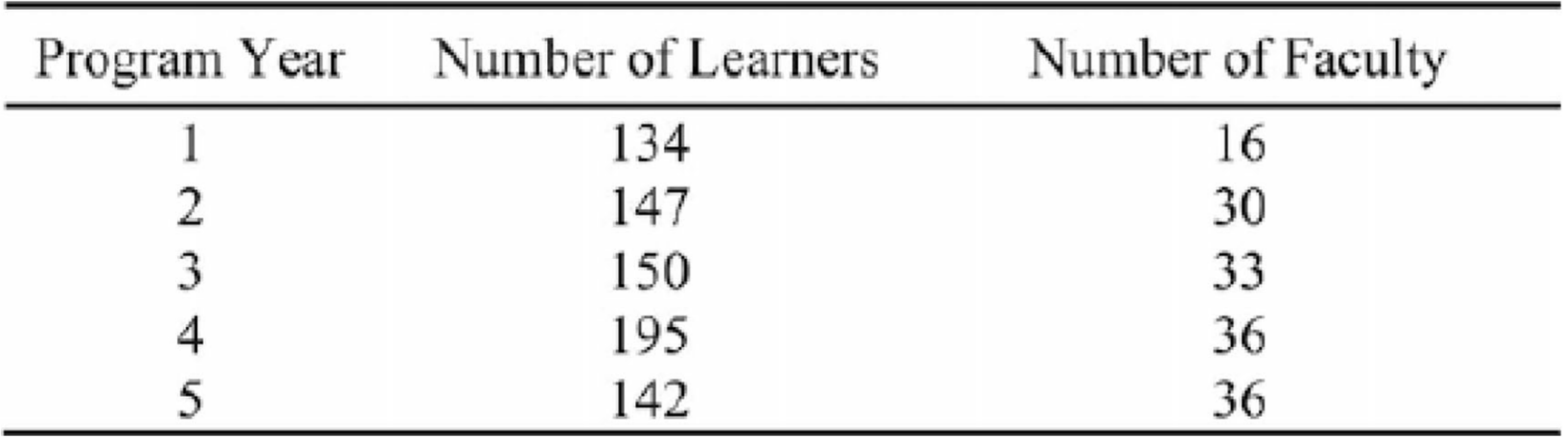
Learner and Faculty Participation by Program Year

**Table 2:**
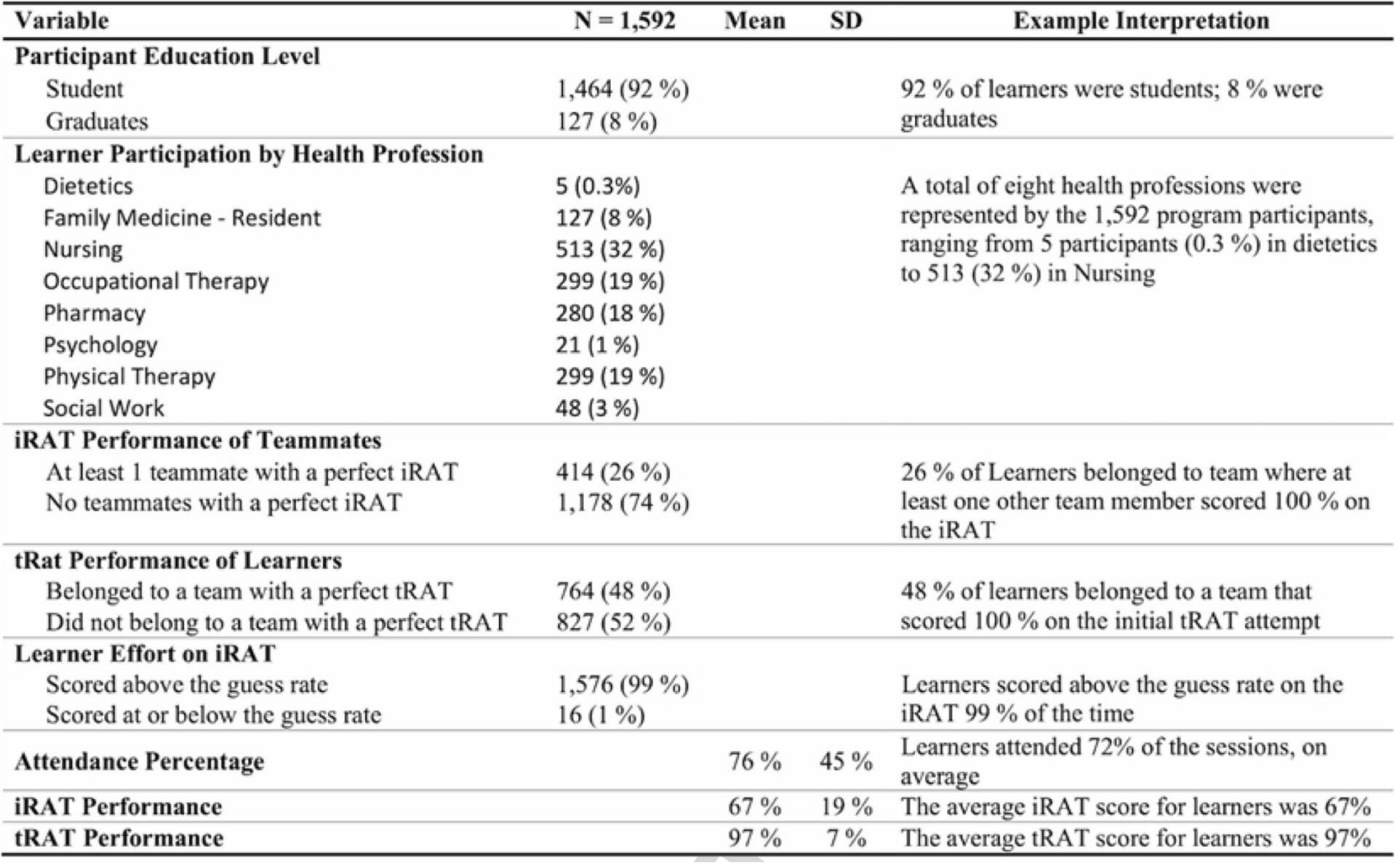
Descriptive Statistics of Learners

## Methods

### Aim

The Geriatrics Champions Program (GCP) was a five-year program from 2011 to 2016. The aim of the program was to create an active-learning platform to train interprofessional learners synchronously for geriatrics care in the workforce. It was funded by a Geriatric Academic Career Award by the Health Resources Services Administration (HRSA-10-228, Grant #K01HP20492, PI: Dr. Shelley Bhattacharya).

#### Content

The GCP was a five-year grant; the first year was program and curriculum building followed by a 24-month curriculum, which repeated twice. Individual programs determined whether the course was required or elective. Each program was represented by a core group of one to two faculty. Faculty were chosen based on interest in IPE and prior collaborations with the team. The core faculty group of 6–10 met monthly to ensure the curriculum met their learners’ needs, to be the content expert of relevant topics and to ensure the content met current healthcare guidelines. Core faculty also helped recruit the cadre of over 100 faculty to be team facilitators each year. Faculty were assigned a small student group to work with through the year.They were instructed to not lead the group, but guide the group if astray, allow for equal participation and spark discussion during lull moments. Faculty participated in required faculty development sessions annually to review the format, goals and responsibilities of each session. The lead facilitator was the Principal Investigator of the grant. She has been the lead facilitator for the six-profession interprofessional geriatrics clinic and brought her IPE skills to this program. Faculty were given a certificate of appreciation each year.

The target audience was learners from Family Medicine residency programs; undergraduate medical clerkships in Family Medicine; Nurse Practitioner tracts for Adult Gerontology and Family Nurse Practitioner programs; Master’s programs in Social Welfare, Occupational Therapy and Psychology; undergraduate Dietetics program; and doctorate programs in Physical Therapy and Pharmacy.

GCP content had thirty objectives divided into eight domains: Special Considerations in Geriatrics Care (Caregiver Burden, Medicare Basics, Function); Medication Management; Cognitive, Affective and Behavioral Health; Complex or Chronic Illnesses in Older Adults; Palliative and End of Life Care; Hospital Patient Safety, Transitions of care; and Ambulatory Care. The domains were guided by the American Geriatrics Society Internal Medicine-Family Medicine (IM-FM) Residency Competencies written in 2010, and expanded to fit each profession’s geriatrics curricular competencies [3]. In addition to the IM-FM competencies, most professions had geriatrics competencies which were reviewed by core faculty and strategically matched to the appropriate domains to allow for all learners to achieve their respective competencies [8–12].

It was expected that program objectives would be better achieved through both dialog with students from other health professions and greater exposure to the GCP curriculum. Accordingly, as seen on Table 3, key variables of interest were the number of different health professions represented by team members in a given session-year and various measures of attendance.

**Table 3:**
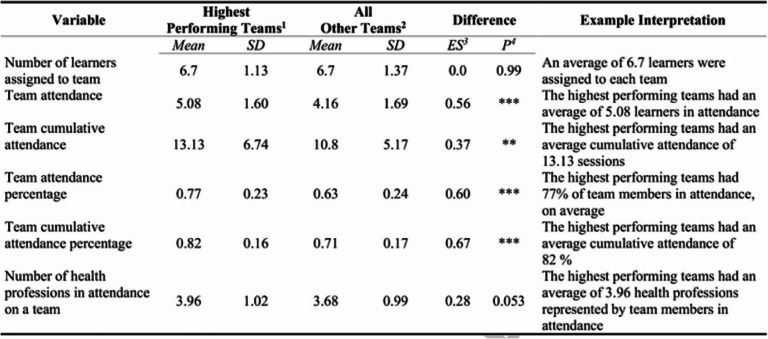
Descriptive Statistics by Team Performance Level

^1^ *N* = 142 teams with 100% on the initial tRAT attempt.

^2^ *N* = 75 teams with less than 100% on the initial tRAT attempt.

^3^ Cohen ‘s D Effect Size (ES) measure for difference between teams scoring 100% (group 1) and less than 100% (group 2) on the initial tRAT attempt.

^4^ * *p* < 0.05; ** *p* < 0.01; *** *p* < 0.001.

The GCP format was a large classroom setting led by the lead facilitator. Learners were divided into 20 small groups of 6–8 learners and 1–2 faculty per group. Learners were intentionally divided into 20 interprofessional teams with at least three professions in each group. Four, 2.5-h sessions were held annually, each focused on one domain. Learners named their teams and stayed within their teams throughout the academic year. The members within each team changed each academic year due to graduation or course completion. Due to the size of the course, the online platform Blackboard® was used.

Finally, after each session, learners were given an evaluation consisting of ten Likert scale and open-ended questions of the value of the session, achieving the session’s objectives, the perceived relevance of IPE to their education and the perceived benefits of IPE to their future practice. These evaluations were regularly reviewed, discussed by program designers to consolidate lessons learned but no formal qualitative analysis was completed.

#### Logistics

Learners were given 60 min of online pre-work before each session which included reviewing articles, online resources, and/or patient cases. Upon arrival to each session, learners were given an online individual Readiness Assessment Test (iRAT) valued at 10 points using the Blackboard® platform. The iRAT consisted of ten multiple choice questions based on the prework material. Learners were shown only their total score, not which items they missed. Immediately following the iRAT (10 min), learners then completed a team Readiness Assessment Test (tRAT) using Immediate Feedback Assessment Technique (IFAT) scratch-off forms. The tRAT valued at 40 points, tasked each team with answering the same iRAT questions, but as a team. Each team member led the team to answer at least one question per session. Each faculty facilitator ensured that all professions’ perspectives were shared for each question. The team IFAT forms had a designated key whereby a star was revealed once the correct answer was scratched off. These scratch-off forms allowed the teams to know if their team answer was correct by selecting the option revealing the star. If an answer was incorrect, they chose an alternate answer until the correct answer was selected. Four points were earned for the correct first attempt; two points if the correct answer was chosen on the second attempt, one point if the correct answer was chosen on the third attempt. The iRAT and tRAT scores were converted to percentages for consistency across scales and compared using Wilcoxon signed rank tests.

Since all students knew their iRAT score, but not which items they got wrong, they could use their team’s reasoning to understand their errors. When all teams completed and submitted their IFAT forms (15 min), the course leader engaged all learners to review any questions as a large group that they found challenging. Teams voiced their support for or against an answer, which encouraged healthy discussion (15 min).

After a short break, the second half of the session consisted of an audio, video or live patient application case relevant to the session’s objectives. Small groups were asked to rotate the roles of a team leader and team speaker to be used for the discussion. Five multiple choice questions were designed to encourage interprofessional collaborative discussion. After ten minutes of discussion, simultaneous reporting was used for each question with teams prepared to defend their answer when others refuted it. This activity allowed learners to apply their pre-work knowledge to the current case, explore their profession’s role in the case, listen to how other professions could help with the patient’s care, and ultimately gather the necessary information to explain their interprofessional care management choice. After the case discussion, five minutes were allocated for online feedback completion.

Points were awarded for each activity towards their team’s final total score. Prizes were given at the end of the academic year to the team with the highest number of points. Participants who attended all four sessions and completed the program evaluation received a certificate of completion for their professional CV.

Materials required to run the program included the following:

1. Geriatrics Champions Program coursework: all prework, coursework, iRAT and case information is available electronically by contacting the corresponding author directly.
2. Interprofessional Faculty: Interprofessional leaders to meet monthly and discuss profession-specific curricula to be included within each of the eight domains. Although the interprofessional faculty together compiled the pre-work for each session, the profession who most closely matched the question’s competencies led the session’s pre-work development.
3. Certificates and Promotion letters: For learners and faculty partners.
4. Online platform: Blackboard®.
5. IFAT forms: commercially available.

While TBL has been associated with better learning outcomes compared to other large group teaching methods, [8, 10] there remains a need to identify factors that make some teams more effective than others [13]. It was hypothesized that tRAT scores would be higher than iRAT scores and that tRAT scores would be higher on teams with more health professions in attendance and on teams with better attendance overall. It was expected that program objectives would be achieved through both dialog with students from other health professions and greater exposure to the GCP curriculum. Key variables of interest include the number of different health professions represented by team members and various measures of attendance (Table 3). Attendance refers to the number of team members present in a session. Attendance percentage refers to the number of team members present as a proportion of the total assigned to the team at the beginning of the year. Cumulative attendance reflects a team’s track record of attendance. It represents the total sessions attended by all team members for all sessions within a year beginning with session one up to and including the current session. Similarly, cumulative attendance percentage represents cumulative attendance as a proportion of the total number of possible sessions attended by all team members.

### Data analysis

The iRAT and tRAT scores were converted to percentages for consistency across scales. Because tRat scores, unlike those for the iRAT, demonstrated ceiling effects and were clustered near top of the score distribution, iRAT and tRAT scores were compared using Wilcoxon signed rank tests. Pearson correlation was used to identify characteristics associated with team performance on the raw tRAT scale. Unpaired t-tests were used to identify pre-post knowledge gains and differences between the highest performing teams and all other teams. The highest performing teams were defined as those scoring 100% on the initial tRAT attempt. This performance threshold served to classify teams by performance group while controlling for the number of tRAT attempts. All statistical analyses used two-tailed tests with a = 0.05 for a 95% confidence level. In all cases, the session-year was the unit of analysis. Effect size interpretations, based on *r* for Wilcoxon signed rank tests and Cohen’s D for unpaired t-tests, reflect the guidelines from Cohen [18].

The analysis plan was adjusted post hoc to exclude both learners who did not make a good faith effort to complete the iRAT (< 1% of all learners) and teams containing another member scoring 100% on the iRAT (26% of all learners, from Table 2). Students are assumed to make a good faith effort if they score above the guess rate, 25% for a multiple-choice exam with four response options per question. It’s important to jointly account for individual and team performance, because participants on teams with another member that scores 100% on the iRAT may have an advantage on the tRAT. If a team member scores 100% on the iRAT and remembers how they responded with certainty, then one team member knows the tRAT answers in advance, potentially biasing the estimate of the difference between iRAT and tRAT scores. Table 4 shows comparisons between teams that got all questions correct on the tRAT first attempt and those that did not on the pooled sample of all teams across all sessions and years (*n* = 217). Tests were repeated on a restricted sample including only those teams with no members that scored 100% on the iRAT (*n* = 142). Results reported are from the restricted sample, though results are robust across both samples.

**Table 4:**
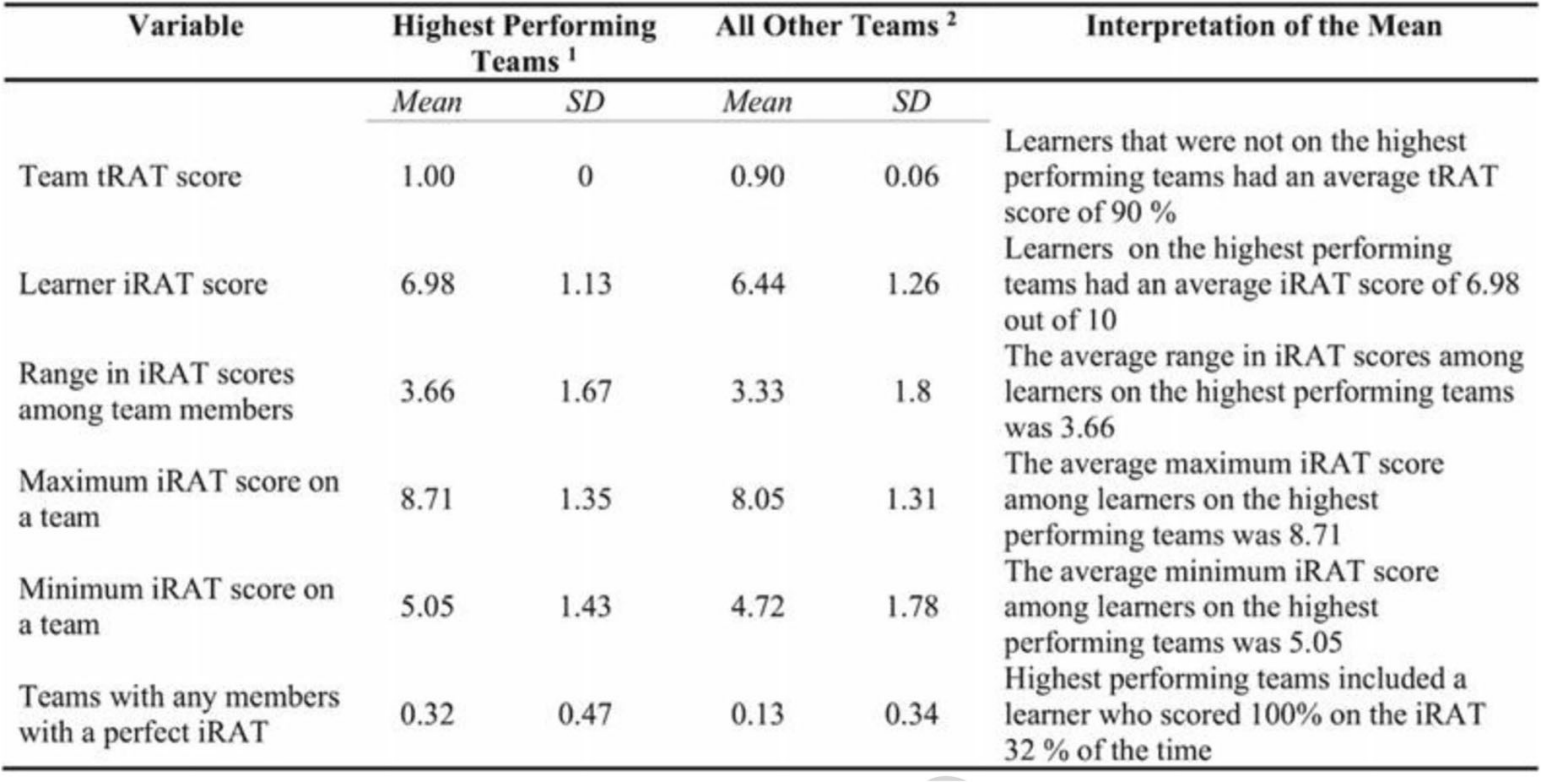
Learner Descriptive Statistics by Team Performance Level

^1^ *N* = 142 teams with 100% on the initial tRAT attempt.

^2^ *N* = 75 teams with less than 100% on the initial tRAT attempt.

## Results

Table 2 provides descriptive statistics of the GCP learners. Over the five years of the Geriatrics Champions Program, iRAT scores $\Big(\ \overline{x}$ = 67%) were lower than tRAT scores $\Big(\ \overline{x}$ = 97%) by 30% (*p* < 0.01, effect size = 0.85). Team performance on tRATs was correlated with cumulative team attendance percentage (r = 0.20, *p* < 0.05), team attendance percentage (r = 0.19, p < 0.05), team attendance (r = 0.20, p < 0.05), average iRAT score among all attending team members (r = 0.23, *p* < 0.01), and maximum individual iRAT score among team members (r = 0.23, p < 0.01). The significance of average and maximum iRAT scores among team members are consistent with recent research finding positive associations between team performance and higher performing individuals on the team [5, 13]. All associations reported above can be considered small in magnitude according to common measures of effect size [7].

**Fig. 1:**
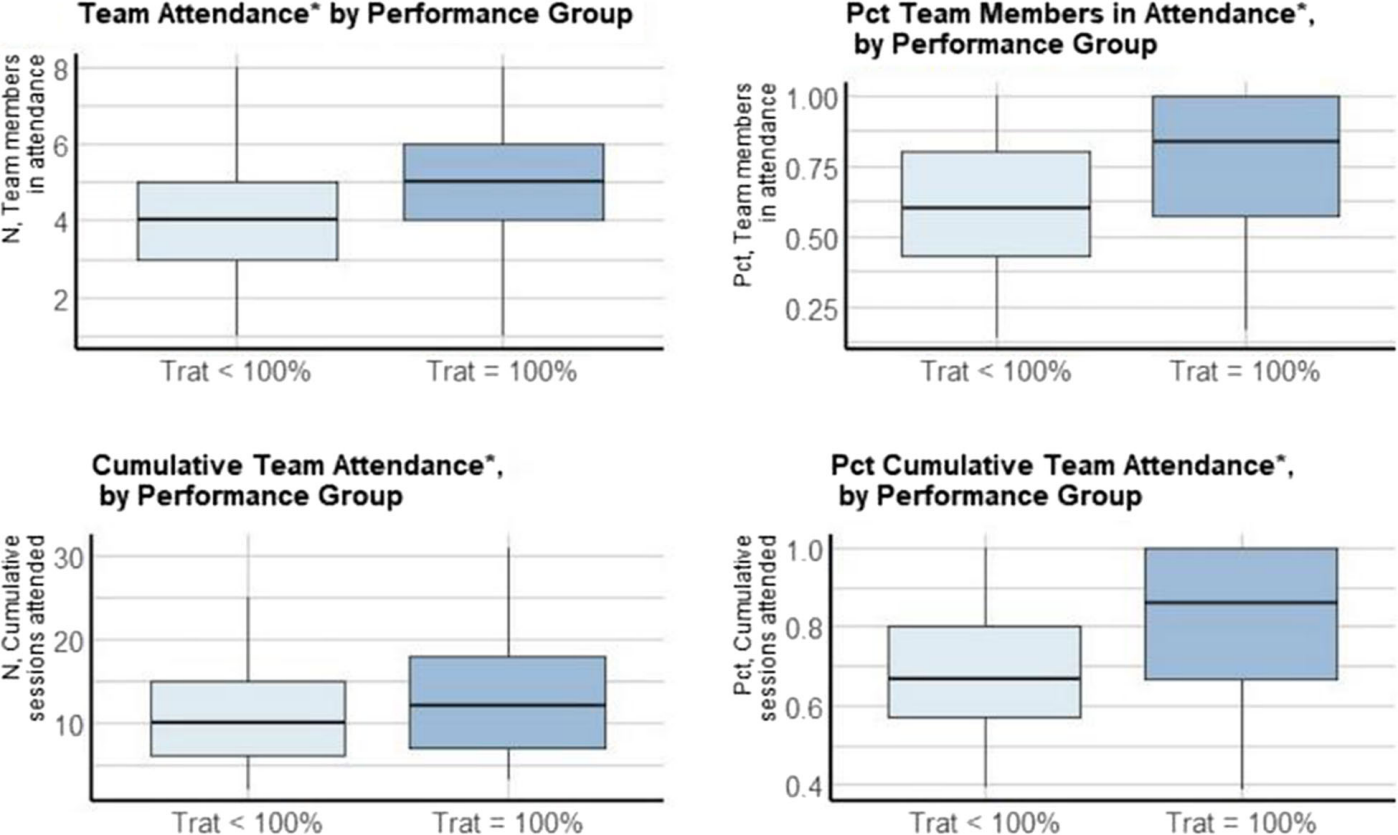
Various Attendance Measures by Team Performance Group

From Fig. 1, the highest performing teams had higher cumulative attendance percentages (t = 4.18, *p* < 0.01), higher cumulative attendance (t = 2.78, *p* < 0.05), higher attendance percentage (t = 3.70, p < 0.01), and a higher attendance (t = 2.86, p < 0.01). From Table 3, the highest performing teams also had an average of 3.96 different health professions represented compared to 3.68 on all other teams (*p* = 0.053, effect size = 0.28).

In the post-program evaluation, written comments (summarized in Table 5) generally indicated that learners felt this program was helpful for their career preparation. One learner wrote, “I really enjoyed participating in the program and hope that future psychology learners will become involved. I discussed this program with a number of faculty members at other institutions during my residency/internship interviews and they were all very impressed with the effort you and the other faculty members have made to help train learners in providing IP care. If I weren’t leaving for my clinical residency/internship this summer I would sign up for next year!” Learners identified improvements on all nine medical knowledge assessment items (Table 6) from pre to post GCP participation.

**Table 5:**
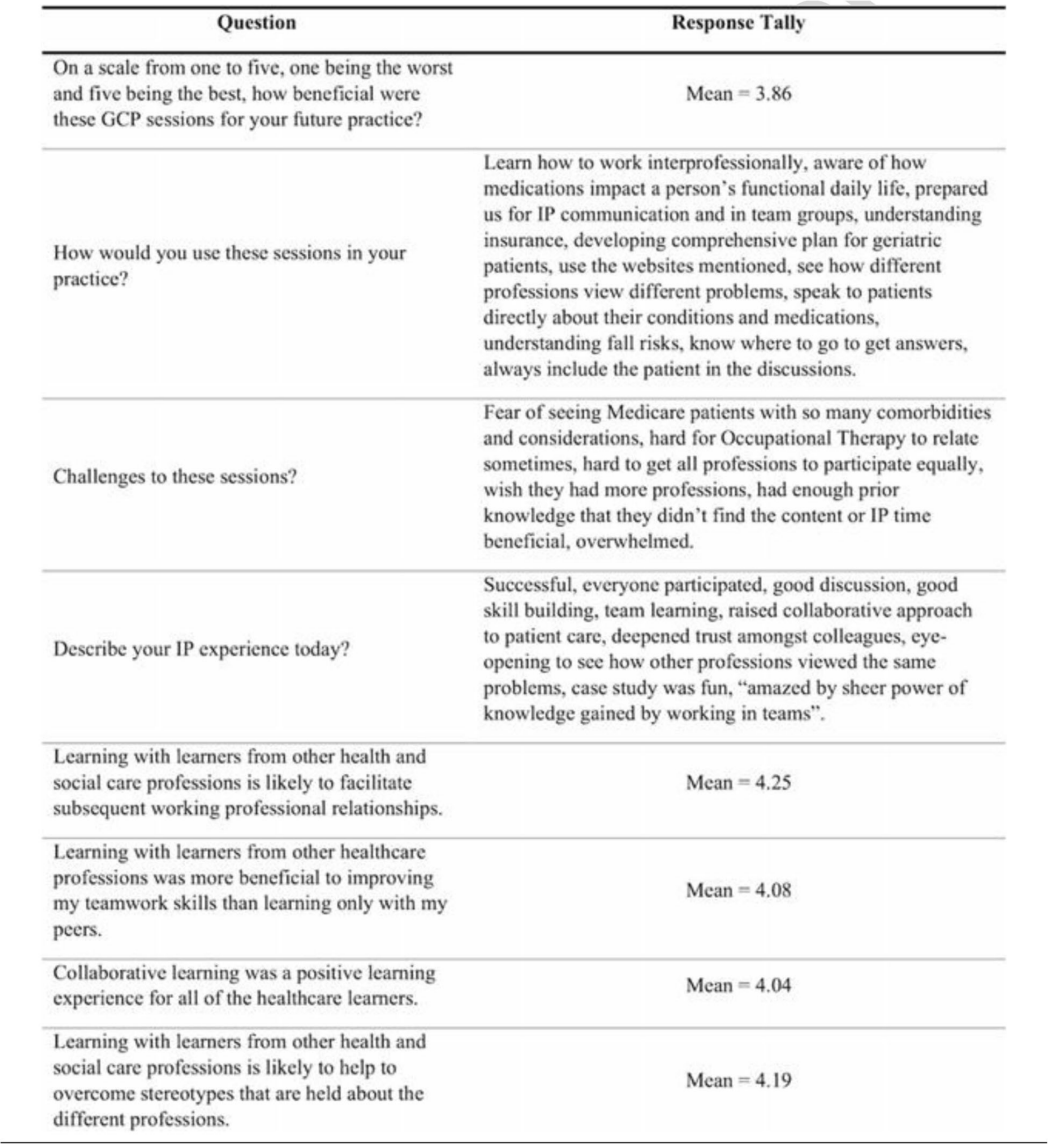
Learner feedback (one as worst and five as best)

**Table 6:**
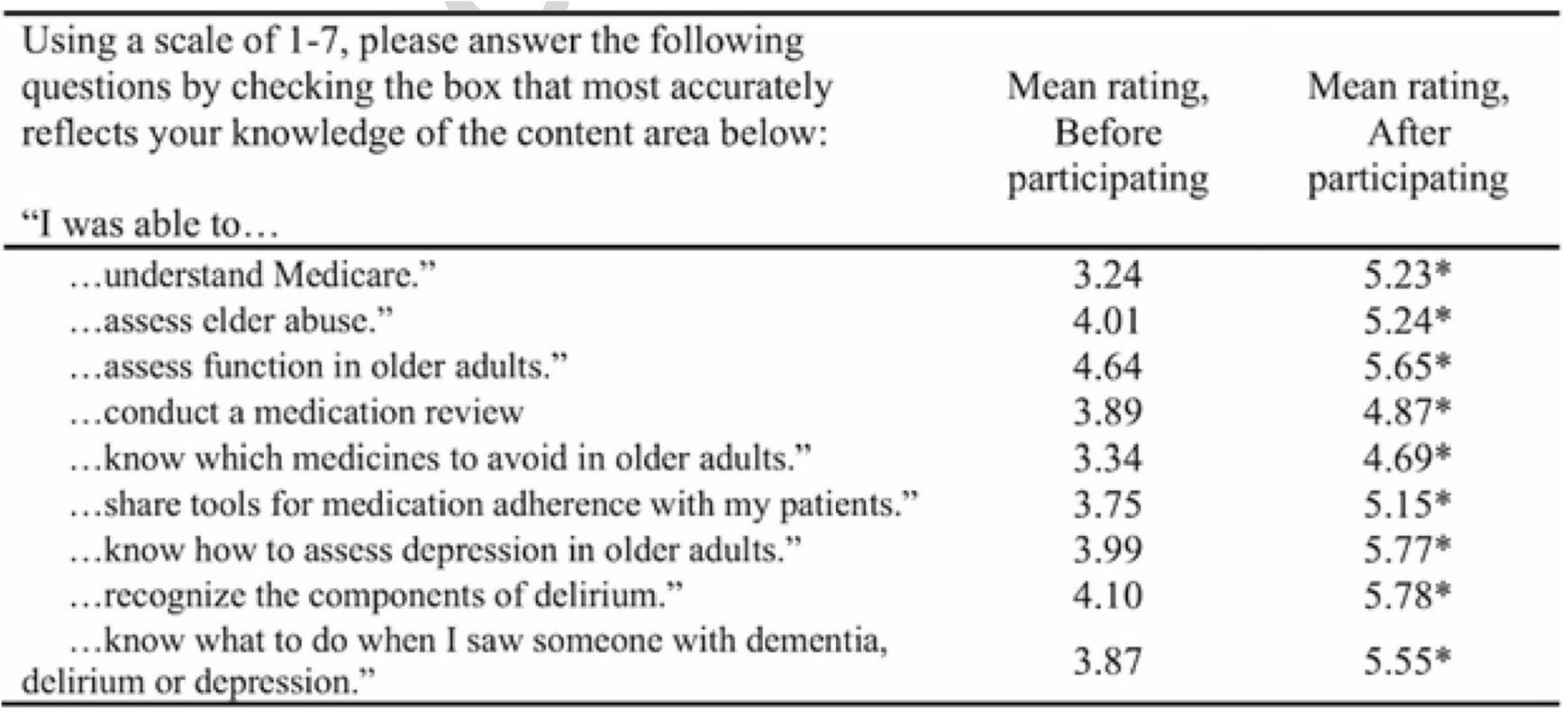
Learner pre-post content knowledge assessment

*Significant difference, *p* < 0.05.

Key:: 1 = strongly disagree; 2 = moderately disagree; 3 = slightly disagree; 4 = neutral; 5 = slightly agree; 6 = moderately agree; 7 = strongly agree

## Discussion

The Geriatrics Champions Program (GCP) was created to unite learners and faculty from seven professions for a workforce preparation course in geriatrics care. It was an interactive IPE program introducing geriatrics domains in a TBL format. Based on the feedback, it was a well-received IPE program that impacted 768 learners and 151 faculty over a five-year period. It will be interesting to see how these interventions impact actual practice in geriatrics care.

A committed faculty contributed to the development and delivery of active learning methods in this geriatrics delivery course. TBL was an effective modality, allowing for efficient delivery of content to a large group annually. Feedback from the learners and faculty was consistently positive and resulted in multiple scholarly poster and podium presentations for learners and faculty.

Learners understood that teams performed better than individuals in geriatrics care. The mean scores for the individual (iRAT) and team (tRAT) assessments differed significantly for each session (*p* < .001) of the Program over the five years. Five-year data demonstrated that scores increased approximately 9–12 points out of 40, or 30%, from the individual to the team assessment. The likelihood of higher performance may have stemmed from interaction and understanding of other professions. The 30% difference remains statistically significant with a large effect size even when the sample is limited to students who both make a good faith effort to complete the pre-work assignments and are not on a team with another participant that scored 100% on the iRAT [7]. Learner feedback on Table 5 and changes in pre-post knowledge on Table 6 demonstrate the value the learners placed on practicing teamwork in geriatrics when in practice. The findings are consistent with previous research that finds individuals perform better in teams than they do alone [13–15].

Higher levels of participation in the GCP are associated with better team performance as measured by tRAT scores. The fact that *percentage* of team members in attendance is associated with tRAT scores suggest that raw attendance may provide an incomplete understanding of the relationship between attendance and team performance.

Prior work by Michaelson and Burlingame suggests that as much as 20–25 h of participation or participation in more than 12 sessions is needed for individuals to fully realize the benefits of learning in groups [19–20]. This study suggests that at least some benefits of the TBL method may be achieved with lower levels of participation. Importantly, both a team’s in-session attendance and track record of past attendance is positively related to team performance. The significance of cumulative attendance, in particular, speaks to the potential latency of the Program’s effect on geriatrics competency attainment. It provides evidence that program benefits may extend beyond individual sessions and last up to at least one year – the duration over which cumulative attendance was measured and over which teams remained unchanged.

Because attendance is largely voluntary, attendance measures allow for variation in team size across sessions. Swanson finds that small groups (defined as those less than five members) perform better in TBL settings [21]. However, this study is consistent with Thompson et al. in finding a positive association between team size and team performance on tRATs [22]. Skilled faculty facilitators may play an important role in supporting the effectiveness of teams with more members, offsetting “social loafing” or “too much talent” type effects [23–24]. Future TBL research would benefit by clearly describing the preparation, skill level and use of faculty facilitators. More research is needed about the role of these facilitators in possibly moderating the effects of team size on learning outcomes. Further TBL studies are needed to confirm effects on learning outcomes [16–17, 25]. TBL requires a paradigm shift in teaching that departs from traditional lecture models. It allows learners to actively acquire new knowledge, and enables the instructor to engage a large classroom environment to help learners apply their newly acquired IPE knowledge to a case scenario.

Multiple lessons can be shared from this interprofessional geriatrics workforce preparation course:

1. Identify IPE gaps in the current healthcare system and ensure your program assists in narrowing or closing the gap. For example, interprofessional geriatric care was a common element missing in our curriculum amongst our four Schools. The GCP served to narrow the gap by providing a platform to teach geriatric interprofessional competencies to learners from all four Schools;
2. Decide which professions you want to include in your program based on availability and needs, and be open to growth as others are interested;
3. Develop a reliable cadre of faculty from each profession that share your vision. Allow their expertise to build your curriculum. Creating a welcoming environment where suggestions and constructive criticism are invited, is key to building lasting partnerships;
4. Meet with your team regularly to share ideas, new content, benefits and challenges;
5. Learn about the components of TBL and how you can incorporate the iRAT, tRAT and Interactive Activity into your IPE activity;
6. Pick a location amenable to small group TBL learning activity discussion;
7. Make the activity a win-win for all, including your learners and faculty. Be flexible in how each profession can be involved and let the faculty determine what fits with their learners and curricula, (e.g. required or optional, all sessions or some sessions);
8. Collaborate with interprofessional faculty when making cases to ensure learners can identify their professional contributions to the case and pose thought-provoking questions to mimic a realistic healthcare system case;
9. Foster a collaborative environment where scholarship is encouraged for learners and faculty;
10. Create a peer evaluation system;
11. Seek funding to support program development;
12. Provide incentives for the learners to participate;
13. Gain institutional support for long-term sustainability.

The GCP was a successful program that incorporated an active learning tool to allow our future interprofessional workforce to engage in geriatrics content they may not have received otherwise. With more programs like these, we can hope that evidence-based, interprofessional geriatrics care will be provided for our older adults. Necessary handouts and content can be obtained via the corresponding author.

## Conclusion

The GCP was an effective program to introduce our budding workforce to the value of interprofessional collaboration in geriatric care. In addition, the TBL format helped identify the benefits of team function. The 30% difference in iRAT – tRAT scores demonstrates to the learners, program leaders and the geriatrics community at large that team efforts consistently outperform individual efforts. Both current and past attendance are important to team performance, consistent with an IPE program where later sessions build upon and expand on content presented at earlier ones. We hope the GCP provides a model that can be replicated at other academic institutions to provide workforce training well-needed to care for our ever-expanding geriatric population.

## Data Availability

Data supporting the results reported in the article can be found on the Principal Investigator’s secure computer’s repository. This data cannot be shared as it would compromise the privacy of the individuals involved. Conditions for access will be limited and only upon approval by the corresponding author. Publicly archived datasets were not used or generated.

## Abbreviations

IM-FM: Internal Medicine-Family Medicine
tRAT: Team Readiness Assessment Test
iRAT: Individual Readiness Assessment Test
IPE: Interprofessional Education
TBL: Team Based Learning
GCP: Geriatrics Champions Program
R1-R3: 1st to 3rd year Family Medicine Resident
MSW: Masters in Social Work
IFAT: Immediate Feedback Assessment Technique
CV: Curriculum Vitae
